# Feature Selection for Motor Imagery EEG Classification Based on Firefly Algorithm and Learning Automata

**DOI:** 10.3390/s17112576

**Published:** 2017-11-08

**Authors:** Aiming Liu, Kun Chen, Quan Liu, Qingsong Ai, Yi Xie, Anqi Chen

**Affiliations:** 1 School of Information Engineering, Wuhan University of Technology, Wuhan 430070, China; aimingliu758@163.com (A.L.); quanliu@whut.edu.cn (Q.L.); qingsongai@whut.edu.cn (Q.A.); yixie2014@126.com (Y.X.); anqichen@whut.edu.cn (A.C.); 2 Key Laboratory of Fiber Optic Sensing Technology and Information Processing, Wuhan University of Technology, Ministry of Education, Wuhan 430070, China

**Keywords:** motor imagery, electroencephalography, brain–computer interface, common spatial pattern, firefly algorithm, learning automata

## Abstract

Motor Imagery (MI) electroencephalography (EEG) is widely studied for its non-invasiveness, easy availability, portability, and high temporal resolution. As for MI EEG signal processing, the high dimensions of features represent a research challenge. It is necessary to eliminate redundant features, which not only create an additional overhead of managing the space complexity, but also might include outliers, thereby reducing classification accuracy. The firefly algorithm (FA) can adaptively select the best subset of features, and improve classification accuracy. However, the FA is easily entrapped in a local optimum. To solve this problem, this paper proposes a method of combining the firefly algorithm and learning automata (LA) to optimize feature selection for motor imagery EEG. We employed a method of combining common spatial pattern (CSP) and local characteristic-scale decomposition (LCD) algorithms to obtain a high dimensional feature set, and classified it by using the spectral regression discriminant analysis (SRDA) classifier. Both the fourth brain–computer interface competition data and real-time data acquired in our designed experiments were used to verify the validation of the proposed method. Compared with genetic and adaptive weight particle swarm optimization algorithms, the experimental results show that our proposed method effectively eliminates redundant features, and improves the classification accuracy of MI EEG signals. In addition, a real-time brain–computer interface system was implemented to verify the feasibility of our proposed methods being applied in practical brain–computer interface systems.

## 1. Introduction

Motor imagery brain–computer interface (BCI) [1] is significant for stroke patients, which can help in the recovery of damaged nerves and play an important role in assisting with rehabilitation training. The application spectrum of the BCI also extends to navigation, healthcare, military services, robotics, virtual gaming, communication, and controls [2,3]. The classification accuracy of motor imagery is of great importance in the performance of BCI systems. As for EEG signal processing, the high dimensions of features represent a technical challenge. It is necessary to eliminate the redundant features, which not only create an additional overhead of managing the space complexity but also might include outliers, thereby reducing classification accuracy [4].

Numerous feature selection approaches have been proposed, including principal component analysis [5], independent component analysis [6], sequential forward search [7], and kernel principal component analysis [8]. These methods can significantly reduce the number of features, but there are still some research difficulties: even if the variance of components meets the requirements, the classification accuracy is still unsatisfactory, probably because they cannot remove redundant features.

Because of the large dimensions of the feature vector, the computational complexity can be increased dramatically, and the redundant features will lead to lower classification accuracy. Aiming at this problem, the genetic algorithm was applied to a motor imagery-based BCI system to reduce the feature vector and eliminate redundant features to solve the high dimension problem [9]. The adaptive regression coefficients, and asymmetric power, spectrum, coherence, and phase locking values were extracted from MI EEG, and the genetic algorithm was used to select feature subsets from the above features, which obtained good results [10]. Other evolutionary algorithms for feature selection include differential evolution [11], artificial bee colony [12], firefly [13], and adaptive weight particle swarm optimization [14].

The essential feature of a genetic algorithm (GA) lies in the global search of data for the group search strategy and evolutionary operator settings. Because of its generalizability, robustness, and easy operation, GAs have been widely used for feature selection [15]. However, they cannot take full advantage of local information, and it takes a long time to converge to the optimal solution. Because biological evolution is essentially a violent search algorithm, differential evolution is easy to fall into the local optimal solution as well as other intelligent algorithms. Adaptive weight particle swarm optimization is an improved algorithm for particle swarm optimization. It can adaptively update weights and ensure that the particles achieve good global search ability and fast convergence speed. The artificial bee colony algorithm has been successfully applied to many problems by simulating the behavior of bee colony intelligence in their search for food to optimize the actual optimization problem. The artificial bee colony algorithm has better global search ability, but its local search ability is weak. The firefly algorithm (FA) was proposed by Xin-She Yang in 2008, which simulates the principle of mutual attraction between individual fireflies to optimize the solution space [16]. The parameters that need to be controlled are relatively few, and the precision optimization and convergence speed are high.

To sum up, the effect of the application of the evolutionary algorithm alone to feature selection is limited, for there is easy entrapment in the local optimum. The evolutionary algorithm and Q-learning algorithm were combined to propose a new feature selection method which is based on the time difference learning method and the firefly algorithm [13].

As a tool of parameter optimization, hypothesis testing, and game theory, learning automata have been applied to the fields of mathematical statistics, automatic control, communication networks, and economic systems. In this paper, we propose a method of combining learning automata (LA) with FA to optimize the parameters of the firefly algorithm, and to avoid getting the local optimum. Common spatial pattern and local characteristic-scale decomposition were used for MI EEG feature extraction. A method of combining LA with FA was employed to select feature subsets, and spectral regression discriminant analysis was used for classification.

## 2. Materials and Methods

### 2.1. BCI Competition Data Set Description

**Figure 1:**
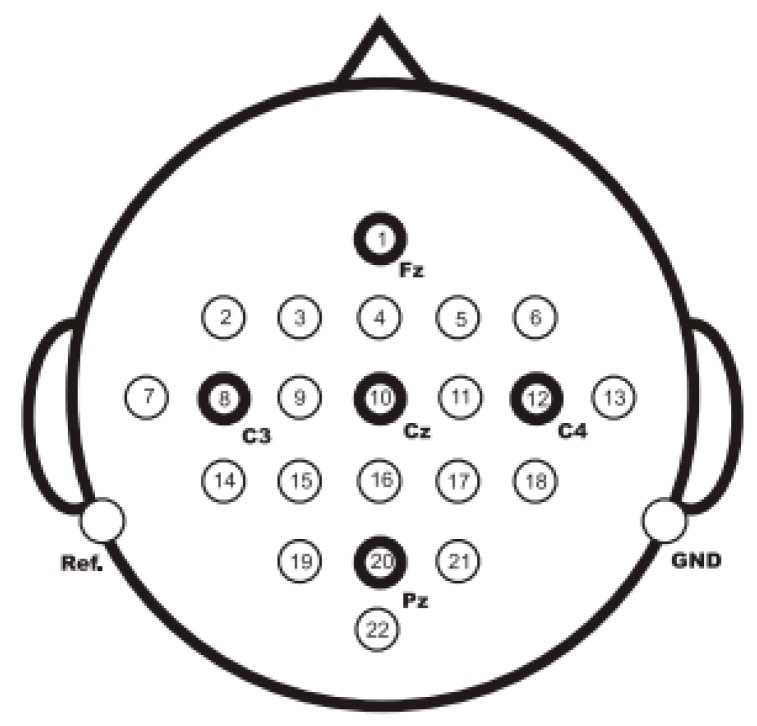
Electrode montage corresponding to the international 10–20 system [17].

The 2008 BCI competition data set 2a provided by Graz University was analyzed to benchmark the performance of our algorithm. Twenty-two Ag/AgCl electrodes were used to record the EEG signals on the scalp. The distribution of all channels is shown in Figure 1 (taken from [17]).

**Figure 2:**
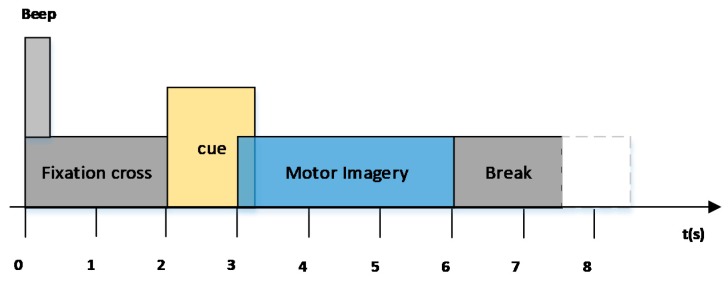
Timing scheme of the paradigm for data set 2a from brain–computer interface (BCI) competition 2008 [17].

The dataset includes four imagination movements: left hand, right hand, both feet, and tongue. Nine subjects recorded EEG data sets on two different days. During the experiment, the subjects sat in front of a computer, reacting according to computer prompts. An experiment lasted 8 s. The experimental timing scheme of the paradigm is shown in Figure 2 (taken from [17]).

The signals were sampled with 250 Hz and band-pass filtered between 0.5 Hz and 100 Hz. The sensitivity of the amplifier was set to 100 μV. An additional 50 Hz notch filter was enabled to suppress power line noise. Each subject’s data set consisted of two parts: the training set and the evaluation set, including the 576 experiments entirely in the data set 2a [17]. 

Before the feature extraction of EEG signals, the signal should be effectively pre-treated to enhance the signal-to-noise ratio and remove artifacts. According to the characteristics of event-related de-synchronization and event-related synchronization, the data were band-pass filtered between 8 Hz and 30 Hz [18] by using a five-order Butterworth band-pass filter.

### 2.2. Real-Time BCI System Design

#### 2.2.1. Experimental Setup

The EEG signals were acquired by using a UE-16B EEG amplifier. It contained 16 channels (Fp1, Fp2, F3, F4, C3, C4, P3, P4, O1, O2, F7, F8, T3, T4, T5, T6) and a low-pass filter with a cut-off frequency of 15–120 Hz. The sampling frequency of the amplifier was 1000 Hz. In our experiment, the 16-channels were selected as the data source, the ground channel was laid at the forehead, and the reference channels were positioned at the mastoids (A1 and A2). The experimenter wore the EEG cap which connected to the signal acquisition device to enable data collection.

Four healthy subjects, two males and two females, right-handed, aged between 22 and 25, and without any neurological diseases, participated in the experiment. In a quiet and dark environment, the subjects kept their muscles relaxed and focused on the experiment to reduce environmental factor and muscle strain interference with the experimental results. Before the experiment, the subjects were given appropriate exercise imagination training to familiarize them with the experimental procedure. All subjects gave their informed consent for inclusion before they participated in the study. The study was conducted with approval from the Wuhan University of Technology.

Four types of motor imagery, including left hand, right hand, double foot and tongue movements, were used as thinking tasks, and the subjects were asked to carry out the corresponding action imagery according to the screen prompt. The training data acquisition experiment procedure is as follows:

- At the beginning of t = 0 s, a beep is generated and the duration of the beep is 1 s. Meanwhile, the screen displays a gray background image that prompts the subject to prepare.
- At t = 2 s, a prompt picture appears on the screen prompting the subject to perform the appropriate MI. The subject’s imaginary thinking activity continues until the prompt picture disappears.
- At t = 6 s, the prompt image on the screen disappears and a gray background image reappears on the screen prompting the subject to rest for two seconds.
- At t = 8 s, a single experiment ends and the next experiment begins.

**Figure 3:**
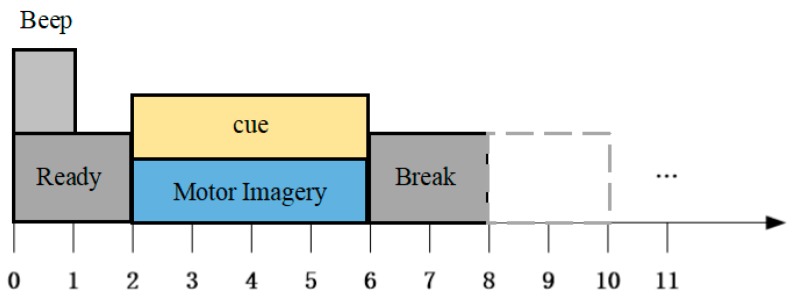
The training data acquisition experiment procedure of a real-time BCI system.

Training data acquisition and experimental process design are shown in Figure 3.

#### 2.2.2. System Design

**Figure 4:**
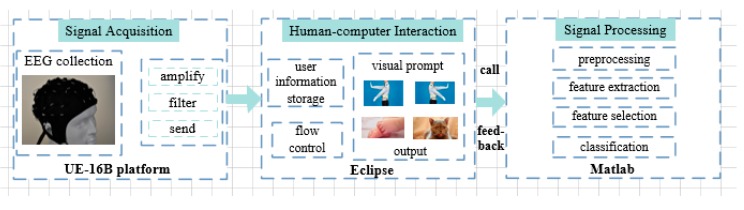
Structure of the real-time BCI system.

In order to verify the feasibility of the proposed algorithm, we designed a real-time BCI system based on MI. The system includes three modules: signal acquisition, signal processing, and human-computer interaction interface. The signal acquisition module collects, filters, and sorts the original electroencephalography (EEG) signal, and then sends the sorted EEG data to the signal processing module through socket communication. The signal processing module processes the EEG data and translates into the corresponding control instructions, and feeds the results back to the user. The human-computer interaction interface module is used for user information storage, experimental flow control, visual prompt, and control instruction output. The overall structure of the real-time BCI system is shown in Figure 4.

**Figure 5:**
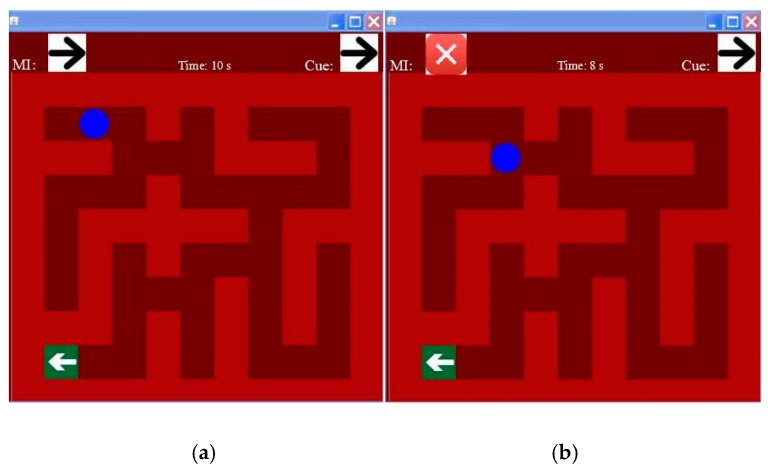
The graphic user interface of the maze game (**a**) The ball moved 1 step from the initial position; (**b**) The ball moved 4 steps from the initial position.

According to the above experimental design, the motor imagery task begins at t = 2 s, and ends at t = 6 s. We segmented the data in this duration into several epochs and conducted a series of experiments to find that the data between 2.5 s to 3.5 s achieved the best performance, so this data epoch was selected for feature extraction and feature selection. Then, a maze game was displayed as seen in Figure 5. The mapping relationship between the motor imagery tasks and movement directions is shown in Table 1.

### 2.3. MI EEG Signal Processing Methods

#### 2.3.1. Feature Extraction

##### Common Spatial Pattern

The common spatial pattern (CSP) is the most commonly used method for MI EEG signal feature extraction [19]. The effect is very prominent, especially for two-class problem of EEG signal classification. We denote the EEG data of trial i for class A, which is a matrix of size N by M. Here N represents the number of channels, and M represents the number of sample points in the time domain of a trial. In theory, there are two classes (namely A and B). By solving the covariance matrix of two kinds of data $R_{A}$ and $R_{B}$, then decomposing the sum of the covariance matrices $R_{A}$, $R_{B}$, the corresponding eigenvector matrix $U_{0}$ and eigenvalue matrix ε are obtained. The whitening transformation matrix P is constructed as P=ε−12U0T.

$R_{A}$ and $R_{B}$ are transformed, and then eigenvalue decomposition is performed to obtain the same eigenvector matrix $U_{A}$ and $U_{B}$. In general, the *m*-th column and the post-m-column eigenvectors can be extracted from $U_{A}$ (or $U_{B}$) simultaneously and combined into a matrix, U, to represent the spatial features of the two types of signals. Then, the spatial filter W is constructed as $W=U^{T}P$.

Using the projection matrix W, the data from each trial, $X_{i}$, can be projected as

(1) $$Z=WX_{i}$$

After CSP projection, *N* rows are selected to represent each trial. Let $Z_{p}\left(p=1,2,\ldots ,N\right)$ be defined as the variance of row p of Z. Then, usually the *p*-th component of the feature vector for the *i*-th trial is computed as the logarithm of the normalized variance as in (2):

(2) $$f_{p}=\log\left[\frac{var\left(z_{p}\right)}{\sum_{p=1}^{N}var\left(z_{p}\right)}\right]$$

The feature vector $f=\left(f_{1},f_{2},\ldots ,f_{N}\right)$ is then used for designing classifiers for motor imagery tasks.

Since this paper designs the four-class MI task classification, the CSP needs to be expanded to satisfy technical requirements. There are two types of commonly used expansion methods: one-to-one and one-to--other. One-to-other was chosen to expand the CSP. Therefore, four projection matrices—$f^{1},f^{2},f^{3},f^{4}$—will be generated for each trial. Each projection matrix is concatenated to form a whole spatial feature vector: $F_{2}$,

(3) $$F_{2}=\left[f^{1},f^{2},f^{3},f^{4}\right]\in R^{1\times 4N}$$

where *N* represents the number of all channels. The signal for each experiment consists of 22 channels of data and 9 channels of ISC component data, so N=31.

##### Local Characteristic-Scale Decomposition

Based on the definition of the intrinsic scale component (ISC), a real value signal x(t)(t>0) can be decomposed into numbers of ISCs by using the local characteristic-scale decomposition (LCD) method in the same manner as [20]. The ISC component’s conditions are to eliminate the situation of riding waves, guarantee the waveform is single, and ensure the smoothness and symmetry of the ISC component waveform. They ensure that the ISC component possesses a single mode between two adjacent extrema and corresponds with the sine curve locally. Therefore, the instantaneous frequency of the ISC has physical significance.

A signal *x*(*t*) is decomposed into n ISCs and a residue $u_{n}(t)$ as

(4) $$x\left(t\right)=\sum_{p=1}^{n}ISC_{p}\left(t\right)+u_{n}\left(t\right)$$

To guarantee that the ISC components meet the definitions, a criterion for the sifting process should be determined. The standard deviation (SD) is adopted as follows:

(5) $$\mathrm{SD}=\overset{T}{\underset{t=0}{\sum }}\left[\frac{{\left|h_{ik}\left(t\right)-h_{i\left(k-1\right)}\left(t\right)\right|}^{2}}{h_{i\left(k-1\right)}^{2}\left(t\right)}\right]$$

Note that time-consumption is still an issue; the maximum contribution of three channels, C3, C4 and Cz, is selected for processing with LCD, and data of each channel is decomposed into three ISCs.

Thus, after processing the EEG signals with LCD, the time-frequency features in a trial are extracted as $F_{1}=\left[f_{11},f_{12}\ldots ,f_{1K}\right]\in R^{1\times KP}$, where K is the total number of ISC components in each experiment, and P is the number of features selected from the ISC component. In our experiment, K=9 and the value of P is set to 20.

##### Feature Fusion

There are two kinds of feature fusion methods: serial feature fusion and parallel feature fusion. Among them, serial feature fusion connects a variety of features after they are normalized, which is simple. In this paper, we employ the serial feature fusion strategy to fuse the features of the spatial and frequency domains.

Assuming the EEG feature vector after processing and serial feature fusion is $F\in R^{1\times \left(KP+4N\right)}$, then

(6) $$\{\begin{array}{c} F=\left[F_{1},F_{2}\right]\\ F_{1}=\left[\frac{f_{11}}{∥f_{11}∥},\frac{f_{12}}{∥f_{12}∥},\ldots ,\frac{f_{1K}}{∥f_{1K}∥}\right]\\ F_{2}=\left[\frac{f^{1}}{∥f^{1}∥},\frac{f^{2}}{∥f^{2}∥},\ldots ,\frac{f^{4}}{∥f^{4}∥}\right]\in R^{1\times 4N} \end{array}\in R^{1\times KP}$$

**Figure 6:**
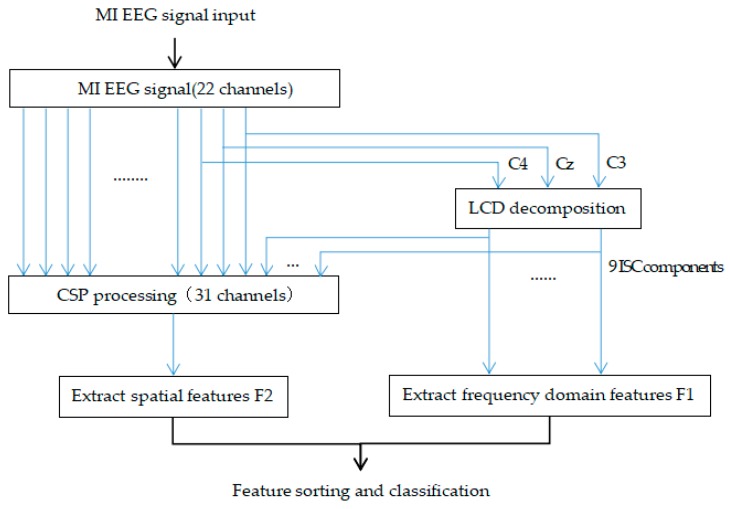
The flow of the proposed feature extraction algorithm, CSP-LCD.

We selected three EEG channels, C3, C4 and Cz, that were believed to be more involved in motor imagery tasks for LCD decomposition [21]. Nine ISC components were obtained and the frequency domain features, $F_{1}$, were extracted. Then, the original signals of the 22 channels were added to the nine ISC components obtained by LCD decomposition, and then the spatial features were extracted from 31 channels as $F_{2}$ by the CSP method. The flow chart of the feature extraction algorithm is shown in Figure 6.

#### 2.3.2. Feature Selection

A brief description of our proposed feature selection scheme (FA-LA) is as follows:

##### Fitness Model Establishment

In order to select a feature subset from the D feature, we set a population with N members. The members of this population were initialized to a vector $\theta_{i}=\left\{w_{i1},w_{i2},\ldots ,w_{iD};w_{ij}\in \left[0,1\right]\right\}$, in which i=1,2,…,N. The Parameter $w_{ij}$ represents the activation thresholds. One feature will be activated when its value exceeds 0.5. The Fitness(i) of the i-th population member was evaluated on the basis of the average classification accuracy ($CA_{v}$) using the validation-set with the spectral regression discriminant analysis (SRDA) classifier which is trained on the Train-set. According to the fitness function formula, Fitness(i)=1CAv, the minimization problem should be carefully studied.

According to the obtained fitness values, the state transition probability matrix was updated.

##### The Firefly Algorithm

The core idea of the FA is that a firefly is attracted by the firefly whose absolute brightness is largest and so updates its location according to the corresponding formula [16]. In the following part, the absolute brightness of the firefly and the attraction between two fireflies are modeled, respectively, and then the updated formula is given.

**Figure 7:**
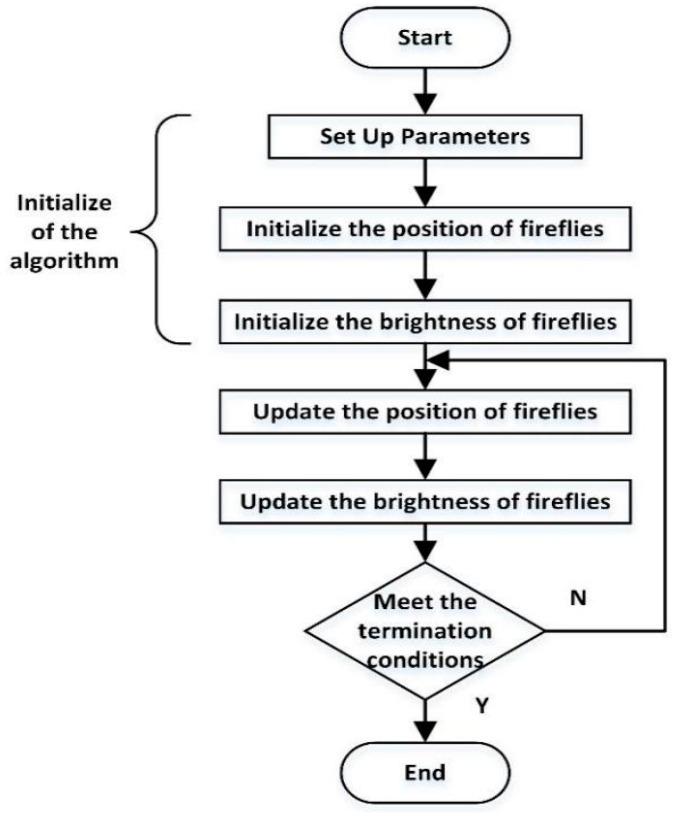
The flow of the firefly algorithm.

The standard firefly algorithm needs to be performed three stages: initialization, firefly location update, and firefly brightness update. The information flow of the firefly algorithm is demonstrated in Figure 7.

In the initial stage, the algorithm parameters are set up and the position of the fireflies is initialized. Then, the position vector of the firefly is fed into the objective function to initialize the brightness of the firefly. According to the brightness of the firefly and the rules of attraction between fireflies, the location of all fireflies is updated in the update stage. In the period of the firefly brightness update process, the new position vector of the firefly is brought into the objective function to complete the update of the firefly brightness. The general termination conditions of the algorithm are as follows: when the algorithm reaches the predetermined number of iterations, and when the algorithm obtains the optimum target value.

##### Adaptive Selection of Parameters for the FA

The state transition probability matrix C is initialized uniformly with 0.05 (due to the lack of a priori information, all values are equally likely) for the parameter γ of FA at 20 quantized levels between (0, 1]. That is $C=\left[C_{1},C_{2},\ldots ,C_{20}\right]$. Thus, the order of C is N×20.

When selecting γ for the first i-th member, we used the roulette method. After generating a random number r, which is between (0, 1], the chosen $C_{j}$ meets the inequality:

(7) $$\overset{j-1}{\underset{m=1}{\sum }}p\left(C_{m}\right)<r<\overset{j}{\underset{m=1}{\sum }}p\left(C_{m}\right)$$

##### Updating the State Transition Probability Matrix

In the m-th generation, we chose $C_{j}$ for the i-th member, then calculated the population fitness value $Fitness\left(i,C_{j},m\right)$, compared with the fitness value Fitness(i,m−1) of the i-th member of the (m−1)-th generation. According to the results of the comparison, we used the linear reinforcement scheme to update the state transition probability matrix $C_{ix}\left(m\right)$ to get $C_{ix}\left(m+1\right)$.

*If* ($Fitness\left(i,C_{j},m\right)<Fitness\left(i,m-1\right)$)

(8) $$\{\begin{array}{c} C_{ix}\left(m+1\right)=\left(1-a\right)C_{ix}\left(m\right),\forall x\neq j\\ C_{ix}\left(m+1\right)=C_{ix}\left(m\right)+a\left(1-C_{ix}\left(m\right)\right),x=j \end{array}$$

Otherwise,

(9) $$\{\begin{array}{c} C_{ix}\left(m+1\right)=\frac{b}{e-1}+\left(1-b\right)C_{ix}\left(m\right),\forall x\neq j\\ C_{ix}\left(m+1\right)=\left(1-b\right)C_{ix}\left(m\right),x=j \end{array}$$

where, a∈[0,1] is the reward feedback factor, b∈[0,1] is the punishment feedback factor, and e is the number of automatic process actions, and was set at 20. Generally, parameters a and b are equal. In our experiment, a=b=0.1 was selected.

##### Pseudo-Code for Feature Selection

- A set of N vectors each with 2D components is randomly initialized between 0 and 1.
- Select the feature subset of each population according to the previous rules.
- Select the feature subset and use the training dataset for training the SRDA, and calculate the fitness value of the feature subset with the validation set according to the fitness function formula.
- Update and reassign the state transition matrix based on the previous validation results according to the fitness value. Population members are updated according to the FA, guided by the fitness values calculated in the previous step.
- If gen < genmax, then go to the second step, otherwise select the member with the best fitness to get the final set of the feature subset.
- The feature subset result obtained in the previous step is used in the test dataset, and the classification result is obtained.

#### 2.3.3. Spectral Regression Discriminant Analysis for Classification

Linear discriminant analysis (LDA) is widely used in feature classification. It is able to maximize the covariance between classes by projecting, while minimizing the covariance within the class. It has been widely used in many areas of signal processing, such as machine learning, data mining, and pattern recognition. However, the computation of LDA involves the eigenvalue decomposition of dense matrices, which is to computationally costly and quite time-consuming.

This paper adopts the spectral regression discriminant analysis classifier. By using spectral graph analysis, SRDA casts discriminant analysis into a regression framework that facilitates both efficient computation and the use of regularization techniques. Specifically, SRDA only needs to solve a set of regularized least-squares problems, and there is no eigenvector computation involved, which is a huge saver of both time and memory.

## 3. Results and Discussion

### 3.1. Offline Data Analysis

As for the 2008 BCI competition data set 2a, there were nine subjects taking part in the experiment, and each subject had two data sets. Session T is the training set, while the session E is the evaluation set. Each of them has 288 trials. In order to lay a good foundation for the classification results, the EEG signal is first band-pass filtered to remove artifacts as described in Section 2.1.

To illustrate the effectiveness of the feature selection algorithm proposed in this paper, the results of SRDA are compared to SRDA with the feature selection algorithm (FA-LA-SRDA). The results are shown in Table 2.

As seen from Table 2, for subjects 1, 3, 5, 6, 7, 8, and 9, FA-LA-SRDA has achieved better results, compared with SRDA. Moreover, the use of the FA-LA algorithm has significantly improved the average accuracy.

In order to further prove the performance of the proposed method, FA-LA was compared with GA and adaptive weight particle swarm optimization (APSO) under the same conditions. The parameters used in competitor algorithms are summarized in Table 3.

**Figure 8:**
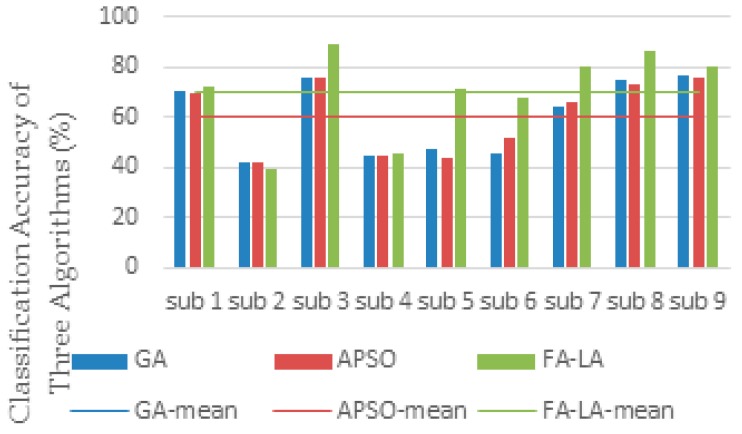
Classification accuracy of the three algorithms: GA, APSO, and FA-LA, for data set 2a from the 2008 BCI competition.

In order to further evaluate the effectiveness of the FA-LA algorithm, a new effective method of combing CSP and LCD for MI EEG feature extraction was proposed, then the FA-LA algorithm was employed for feature selection. The classification accuracy and the number of selected features were compared with the other two feature selection methods, GA and APSO. The comparison of the performance metrics of the three algorithms is given in Table 4 and Figure 8, where the classification accuracy (CA) and the number of selected features (FS) of each subject are the two critical metrics.

By comparing the values of the CA of the three algorithms in Table 4 and Figure 8, the FA-LA algorithm achieves the highest classification accuracy for all subjects, except subject 2, compared with the GA and APSO algorithms. The average classification accuracy is also the highest among the three algorithms. The average number of features selected using FA-LA and APSO are similar: 159 and 161 respectively. Compared with the average number of 216 features using the GA, the number of features chosen by this method is significantly reduced.

When comparing the results of feature selection and classification in both cases, it can be seen from Table 4 that, although the number of features selected based on CSP and LCD is relatively large, the average classification accuracy of the three algorithms is higher than that based on CSP classification. The results show that combination with the LCD algorithm to extract the time-frequency domain features can extract more useful information, so as to improve the classification effect. In the three feature selection algorithms, the average classification accuracy of the proposed FA-LA algorithm is still the highest, which also verifies the advantage of the algorithm’s performance.

### 3.2. Comparison to Previous Work

Table 5 summarizes the comparison of classification accuracy (CA) and the kappa score (K) of the proposed method with the existing multi-class methods for each subject in data set 2a of BCI competition IV. We applied a 10-fold cross-validation procedure for the proposed method.

As seen from Table 5, for the method proposed in the literature [22], the effect of subject 2 is the best, but the average classification accuracy is the worst of the four methods. Although an improved classification method is proposed in [22], the effect is not obvious and acceptable. In [23], pre-processing and channel selection was performed for the EEG signal. Subjects 5 and 7 achieved the best performance, but the average classification accuracy was around 66.6% and the kappa score was 0.55, which ranks third out of the proposed methods. The average classification accuracy and kappa score of the FA-LA method achieved a mean performance of 70.2% and 0.6 respectively, which is same as the score in the literature [24]. Subject 3 achieved the best classification accuracy, 88.89%, in all subjects. The proposed method outperforms the reference methods. It proves that the proposed feature selection algorithm can effectively improve classification accuracy.

### 3.3. Real-Time Data Analysis

In the real-time experiment, according to the parameter setting of the UE-16B EEG amplifier in the data acquisition platform (as described in Section 2.2), the original EEG signal collected by the EEG cap will be roughly filtered, and then the EEG signal output from the amplifier will be subject to band-pass filtering such as off-line data preprocessing.

**Figure 9:**
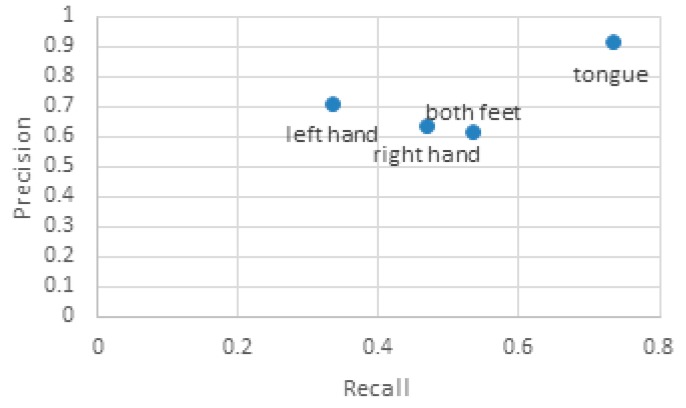
The precision and recall rate of the classification results of four-class motor imagery tasks: both feet, left hand, right hand, and tongue, for real-time MI EEG signals.

Each subject completed 15 trials of experiments for each class of motor imagery tasks; a total of 60 trials. F-measure was used to analyze the performance of the proposed methods in real-time BCI systems. It is calculated as: $F=\frac{2\;*\;P\;*\;R}{P\;+\;R}$. The average precision (P), recall (R) rate, and F-measure of each class of movement is illustrated in Table 6 and Figure 9.

In general, it is expected that the recall rate and precision of the results be closer to 1, but the two are sometimes contradictory, so F-measures can be used to evaluate the results comprehensively. From Table 6 and Figure 9, it can be seen that the effect of the tongue is the best of the four-class MI tasks, with higher precision and recall rates than the other three, especially with a precision of 0.92, which shows that this class of MI task is not easily misjudged; other tasks are not easily mistaken for tongue task. The precision of left hand, right hand, and foot tasks is still relatively high, but the recall rate is not satisfactory. The precision of the left hand can reach 0.7, but its recall rate is the lowest of the four classes, indicating that this task of MI can easily be mistaken for other tasks.

In order to further and more intuitively verify the performance of the feature selection algorithm proposed in this paper, the experimental results were compared with the genetic algorithm and the adaptive weight particle swarm optimization algorithm. After four subjects were adapted to the system, 100 experimental data were collected from the system. In total, 75% of the experimental data was selected as training data and 25% was selected as test data. Each group of data was processed 10 times by selecting different training sets and test sets, and the final results were averaged, as shown in Table 7.

According to the real-time experimental results as seen in Table 7, the FA-LA algorithm proposed in this paper had obvious effects on all four subjects. The number of features selected is also the least of the three algorithms, except for the subject 2. On the whole, the number of features selected by the genetic algorithm is the largest, with an average value of 332, while the number of features selected by APSO and FA-LA is significantly reduced by 301.5 and 296, respectively. In the above-mentioned subjects, the number of features selected for subject 2 was the highest, and the classification efficiency was the highest. Subject 1 underwent the least number of experiments, and the classification accuracy of each feature extraction method was low. This shows that motor imagery can be better trained to produce the appropriate instructions and get better classification results. The overall trend is consistent with the results of the competition data, and the validity of the feature algorithm is verified again.

The execution time of completing the real-time maze game is a reasonable choice to verify the real-time performance of the MI signal processing algorithm in this paper. Table 8 shows the total time for four subjects to complete five trials of maze games, respectively.

The time for a single data epoch is 1.1 seconds. Within this time, EEG data is collected and processed to obtain the final results. If the classification results are correct, the ball will move in the direction indicated by the picture, and if the classification results are wrong as shown on the right of Figure 5, the ball will stop. The subject prompts the ball to move to the position of the green arrow. There are 19 steps in total to complete the maze game.

Because of individual differences, in the training data collection section, each subject’s analysis results are not the same, and the classification model is also different. The game process is long and the classification accuracy of a certain step on the way is too low to affect the mood of the subjects, thus affecting the overall performance of the game. The subjects completed the maze game in a total time of around 50 s, and the average time of each step was about 2.6 s, so the overall real-time effect is still acceptable.

For the parameters of CA and FS, the FA-LA feature selection algorithm, to a certain extent, improved classification performance and achieved the best performance in comparison with the other two algorithms. Thus, it shows that the feature selection method FA-LA proposed in this paper can balance the number of features and classification accuracy effectively, and the final maze game also confirmed that the algorithm achieves good real-time performance.

## 4. Conclusions

In this paper, we proposed a novel feature selection method based on the firefly algorithm and learning automata for four-class motor imagery EEG signal processing to avoid being entrapped in the local optimum. After feature extraction using a method of combining CSP and LCD from the EEG data, the proposed feature selection method FA-LA is used to obtain the best subset of features, and the SRDA is utilized for classification. Both the fourth brain–computer interface competition data and real-time data acquired in our physical experiments were used to confirm the validation of the proposed method. Experimental results show that the proposed FA-LA further improves the recognition accuracy of motor imagery EEG, mainly decreases the dimensions of the feature set, and is capable of operating in a real-time BCI system.

In future work, we will further improve the signal preprocessing method to remove eye artifacts in the EEG signal. How to determine the existence of eye artifacts, and how to adaptively remove the eye signals, are critical research issues. The future goal is to apply this method to the real-time control of devices such as robotic arms, which will be helpful in the rehabilitation of patients with functional paralysis.

## Figures and Tables

**Table 1:** The mapping relationship between the motor imagery tasks and movement directions.

Classification Labels	Motor Imagery	Game Move
0	Both feet	Up
1	Left hand	Left
2	Right hand	Right
3	Tongue	Down

**Table 2:** Comparison of classification accuracy (%) of spectral regression discriminant analysis (SRDA) and firefly algorithm (FA)- learning automata (LA)-SRDA for data set 2a from the 2008 BCI competition.

Subject	1	2	3	4	5	6	7	8	9	Mean
**SRDA**	59.72	39.93	72.22	52.08	35.76	41.67	60.07	73.96	76.74	56.91
**FA-LA-SRDA**	**72.22**	38.89	**88.89**	45.83	**70.83**	**68.06**	**80.56**	**86.11**	**80.56**	70.2

**Table 3:** Parameter settings in competitor algorithms.

**GA**	N = 50, Cross-over Probability = 0.9, Mutation probability = 0.04
**APSO**	N = 50, Acceleration Coefficients $c_{1}=c_{2}=2$, Inertia Factors $w_{\max}=0.9$, $w_{\min}=0.6$
**FA-LA**	N = 50, Minimum Attractiveness $\beta_{\min}=0.3$, random vector coefficient α=1

**Table 4:** Comparison of classification accuracy (CA, %) and the number of the features selected (FS) between the three algorithms: GA, APSO, and FA-LA, for data set 2a from the 2008 BCI competition.

Subject	GA		APSO		FA-LA	
	CA	FS	CA	FS	CA	FS
1	70.03	226	69.45	155	**72.22**	160
2	41.56	251	42.14	149	38.89	158
3	75.34	256	75.36	159	**88.89**	161
4	44.34	214	44.93	156	**45.83**	156
5	47.51	235	43.92	155	**70.83**	162
6	45.28	261	51.76	181	**68.06**	161
7	63.75	165	66.26	156	**80.56**	159
8	74.37	160	73.14	172	**86.11**	165
9	76.49	174	76.11	165	**80.56**	153
**Mean**	59.85	216	60.34	161	70.2	159

**Table 5:** Comparison of classification accuracy (CA, %) and kappa score (K) between the four methods: HSVM, SVM, TSLDA, and our proposed method, for data set 2a from the 2008 BCI competition.

Subject	HSVM [22]		SVM [23]		TSLDA [24]		Proposed Method	
	CA	K	CA	K	CA	K	CA	K
1	68.90	0.59	59.29	0.46	**80.50**	0.74	72.22	0.63
2	**66.70**	0.56	59.29	0.46	51.30	0.35	38.89	0.19
3	72.50	0.63	57.5	0.43	87.50	0.83	**88.89**	0.85
4	44.80	0.26	55.36	0.40	**59.30**	0.46	45.83	0.28
5	40.70	0.21	**76.07**	0.68	45.00	0.27	70.83	0.61
6	38.50	0.18	56.07	0.41	55.30	0.40	**68.06**	0.57
7	75.40	0.67	**83.93**	0.79	82.10	0.76	80.56	0.74
8	72.20	0.63	76.07	0.68	84.80	0.80	**86.11**	0.81
9	62.70	0.50	75.71	0.68	**86.10**	0.81	80.56	0.74
**Mean**	60.30	0.47	66.60	0.55	70.20	0.60	70.20	0.60

**Table 6:** F-Measure statistics of the classification results of four-class motor imagery tasks: both feet, left hand, right hand, and tongue, for real-time MI EEG signals.

Parameter	Both Feet	Left Hand	Right Hand	Tongue
P	0.62	0.71	0.64	0.92
R	0.53	0.33	0.47	0.73
F-measure	0.57	0.45	0.54	0.81

**Table 7:** Comparison of classification accuracy (CA, %) and the number of features selected (FS) between the three algorithms: GA, APSO, and FA-LA, for real-time MI EEG signals.

Subject	GA		APSO		FA-LA	
	CA	FS	CA	FS	CA	FS
1	48	316	48	294	58	291
2	68	385	72	314	75	298
3	58	300	56	301	62	302
4	56	327	60	297	68	293

**Table 8:** Execution time for different subjects conducting different trials of a maze game.

Subject	Experiment 1	Experiment 2	Experiment 3	Experiment 4	Experiment 5	Mean
1	51.7	44	45.1	42.9	38.5	44.44
2	70.4	49.5	68.2	58.3	45.1	58.3
3	53.9	47.3	68.2	49.5	40.7	51.92
4	38.5	45.1	56.1	38.5	53.9	46.42
